# In vivo Susceptibility of *Plasmodium vivax* to Chloroquine in Southeastern Iran

**Published:** 2012

**Authors:** A Heidari, H Keshavarz, S Shojaee, A Raeisi, S Dittrich

**Affiliations:** 1 Dept. of Pathobiology, School of Medicine, Alborz University of Medical Sciences, Karaj, Iran; 2 Dept. of Medical Parasitology and Mycology, School of Public Health, Tehran University of Medical Sciences, Tehran, Iran; 3 Center for Research of Endemic Parasites of Iran (CREPI), Tehran University of Medical Sciences, Tehran, Iran; 4 Dept. of Medical Entomology and Vector Control, School of Public Health, Tehran University of Medical Sciences, Tehran, Iran; 5 Molecular Biology Laboratory Lao Oxford Mahosot Welcome Trust Research Unit, Vientaine, LaoPDR

**Keywords:** Malaria, *Plasmodium vivax*, Chloroquine, Iran

## Abstract

**Background:**

*Plasmodium vivax* is the predominant species causes of malaria with about 90% total annual reported malaria in Iran. This study conducted to determine the susceptibility of *Plasmodium vivax* isolates to chloroquine in Sistan and Balochistan Province, southeastern Iran.

**Methods:**

A total 270 subjects with symptomatic malaria and confirmed *P. vivax* infection completed the designed 28-day in vivo study. The thick and thin film blood smears were screened for malaria parasites by microscopy. The nested PCR was applied using the *Plasmodium* 18 subunit ribosomal ribonucleic (Ssr RNA) genes for detecting mixed infections and diagnosis of parasites in the samples with low parasite on days 0, 5, 6, 7, and 28.

**Results:**

*P. vivax* was cleared in 15%, 50%, 95%, and 100% of patients on days 1, 2, 3, 4 respectively by microscopy assessment. Six patients were exhibited specific *P. vivax* band in nested PCR on day 5. No recurrence was observed on days 7, 14 and 28. Mean (±standard deviation) parasite clearance time was 2.41 (±0.8) days.

**Conclusion:**

*P. vivax* is still susceptible to chloroquine in Southeatern Iran. This finding is compatible with results of neighboring countries Pakistan and Afghanistan.

## Introduction

*Plasmodium vivax* (*P. vivax*) is the main cause of malaria in Asian, Central and South American countries (1). Meanwhile it is actually coming back to Europe with recent report from Greece (2). About 70-80 million symptomatic malaria cases due to *P. vivax* occur in the world annually (3). Since six decades ago, chloroquine has been used for treatment of human malaria in the world. Resistance to chloroquine occurred in *P. falciparum* very soon after extensive use of this agent in endemic areas around the world. Thereafter, the emergence of chloroquine resistant *P. vivax* was reported for the first time in Australian soldiers returning from Papua New Guinea (4). Consequently; chloroquine resistant *P. vivax* was observed in Myanmar (5), southeastern Turkey (6), Ethiopia (7, 3), Republic Korea (8), Indonesia (9, 10), India (11) and with reports also from geographical regions in Southern and Central America (4, 12). These reports underline again the serious concerns about emerging resistance in *P. vivax* isolates to chloroquine in new regions of the world. However, chloroquine seems still a suitable drug in Afghanistan (13), Azerbaijan (14) and Thailand (15). *P.vivax* is the predominant causative species of malaria with about 90% of the total annual reported cases of malaria in Iran and both were *P.falciparum* and *P.vivax* have been reported since 1921 (16).

*Plasmodium falciparum* resistant to chloroquine and sulfadoxin premethamin (S/P) has been reported in Iran (17, 18). Furthermore, S/P and artosonate combination is currently administered for treatment of this infection. While the decreased susceptibility of *P. vivax* to chloroquine has been reported in clinical studies in south of Iran (17, 19, 20), chloroquine has been the main drug of choice for treatment of patients infected by *P. vivax*. Therefore, assessment of susceptibility of *P.vivax* to chloroquine is an important public health priority in endemic countries such as Iran.

In recent years, a decrease in reported cases of malaria has been occurred in Iran. The statistics related to years 2008 and 2009 are 11,460 and 6,122 malaria patients, respectively (21). Immigration from, Afghanistan and Pakistan, two malaria endemic neighbor countries, resistance of vectors to insecticides, socioeconomic situation and emergence of P. falciparum species which are resistant to some anti-malaria drugs have impeded malaria control in this part of Iran (22, 23).

In order to ensure good patient care and to support the malaria control efforts of the Health Ministry in Iran, we performed a susceptibility study for *P. vivax* in the southeast of Iran following clinical reports of resistance from neighboring countries.

## Materials and Methods

### Study area

Sistan and Baluchistan Province is located in southeast of Iran and possess common borders with Pakistan and Afghanistan. The highest numbers of malaria infection in Iran have been reported from this province during the past decade. *Anopheles (A) stephancy*, *A. culcificis*, *A. fluviatalis, A. superpictus and A. pulcherrimus* are the main malaria vectors in the area (24). *P. falciparum* resistance to chloroquine was reported for the first time in this province in 1983 (25). The malaria transmission is unstable with two peaks from May to August and from October to November. The study sampling was carried out between May and November 2010 at the health centers in Sistan and Baluchistan province. These centers provide malaria diagnosis and treatment services free of charge. Both autochthonous and imported malaria have been reported in the area. Imported malaria originated from Pakistan and Afghanistan.

### Study samples

A total of randomly 285 subjects with symptomatic malaria and confirmed *P. vivax* infections participated in the study. All of them were referred to the health centers of Chabahar distinct located in Siststan and Baluchistan Province to receive required treatments. However, 15 patients were lost to follow up in various stages of the study mostly due to traveling or mix (*P. vivax and P. falciparum*) infection.

Age of the patients ranged between 2 and 60 years. Chloroquine was administrated to all subjects during 3 days (10mg/kg on days 0 and 1and 5mg/kg on day 2). According to the Iran national drug policy, primaquine (PQ) is given during 8 weeks for preventing relapses. Since half-life time of chloroquine extends nearly 30 days and during this time it can eliminate emergent parasites (Baird 2009, Naing 2010), thus PQ administration was postponed to day 28. Inclusion criteria for the registered malaria patients were patients who aged more than 1 year old, positive for *P. vivax* mono infection with parasite density above 250/µl, and fever or history of fever during 48 hours prior to the time of recruitment. Patients with the following conditions were excluded from the study: presence of clinical condition requiring hospitalization, presence of severe malnutrition, pregnancy, chronic infectious diseases, and concomitant febrile illnesses. All patients were followed up for 28 days according to the WHO *in vivo* test protocol (26). The patients were visited on days 1, 2, 3, 4, 5, 6, 7, 14, 28. Auxiliary temperature was measured to detect fever and thick and thin blood slides were collected on the above-mentioned days.

The treatment failure was defined as clinical deterioration due to *P. vivax*, which required hospitalization in presence of parasitemia, presence of parasitemia and auxiliary temperature of ≥ 37.5 at any time between days 3 and 28, and presence of parasitemia between days 7 and 28 irrespective of clinical conditions. Blood samples were taken on days 0, 5, 6, 7, 28 and stored at -20°C until DNA extracting for nested PCR. The study protocol was approved by the Tehran University of Medical Sciences, Iran. The consent obtained from all participants or their parents.

### Laboratory investigation

Thick and thin film blood smears were prepared and stained with 3% Giemsa. Parasite density was determined for every positive thick film by counting the number of parasites against 200 white blood cells and was multiplied by 40 to obtain the number of parasites per mm^3^ of blood. Genomic DNA was extracted from the 200µL blood samples using a QIA quick PCR purification kit (Quigen, Germany) according to the manufacturer^’^s protocol. The nested PCR was applied using the *Plasmodium* 18 sub-unit ribosomal ribonucleic (Ssr RNA) genes for detecting mix infections and diagnosis of parasites in the samples with low parasite (less 10 P/µl). Primers and detail PCR amplifying method have previously been described (27). Negative and positive controls were used in each set of experiments. The amplified products were run in 2% agarose gel electrophoresis and stained by ethidium bromide for visual detection bands by ultraviolet transillumination.

## Results

**Fig. 1:**
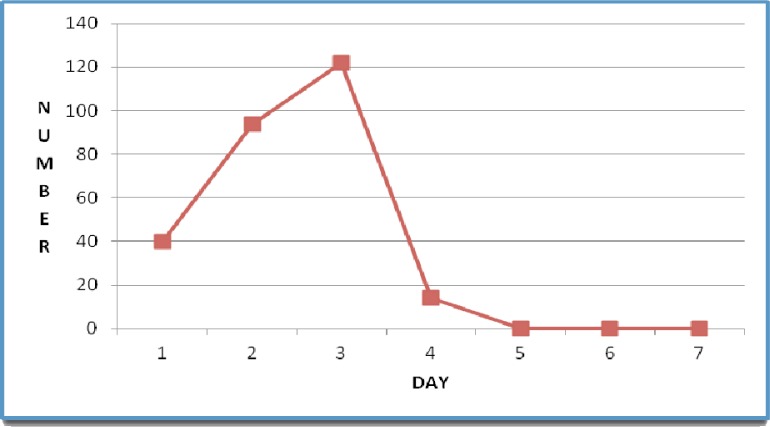
Parasite clearance time during the 28 days of patients follow up after chloroquine treatment in infected patients with *Plasmodium vivax* in southeast of Iran

Out of 270 patients who completed the 28-day follow-up, 180 individuals (66.6%) were male. chloroquine was well tolerated and no patient reported vomiting within 1h of drug administration. Fever resolved on the first day in all subjects. According microscopy findings *P. vivax* was cleared in 15%, 50%, 95%, and 100% of patients on days 1, 2, 3 and 4, respectively (Fig. 1). No recurrent infection was observed on days 7, 14 and 28.

**Fig. 2:**
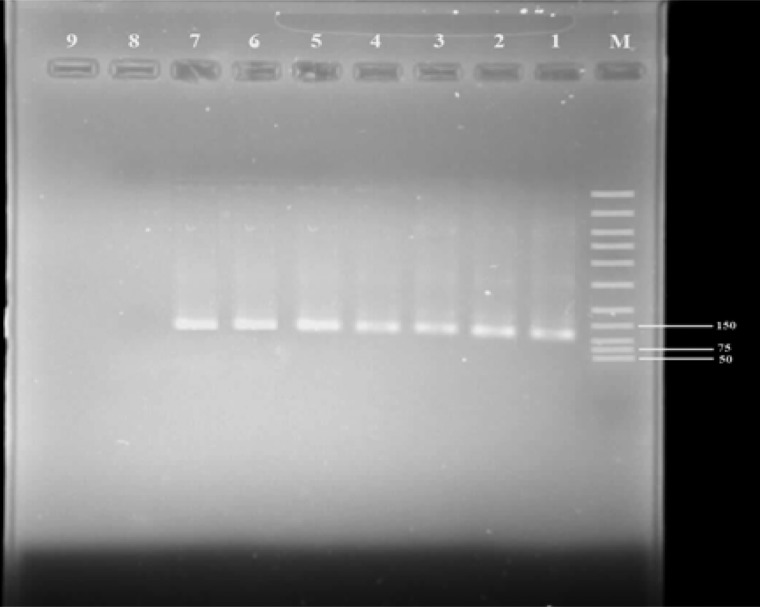
Gel photograph showing nested PCR amplified products from different *Plasmodium vivax* infected isolates in southeast of Iran on day 5. The DNA marker is shown on the right (Lane 1). Lane2 to 7 are Patients *Plasmodium vivax* isolates. Lane 8 and 9 are *Plasmodium falciparum* and negative controls respectively

The PCR product was consider positive when a band of size of ∼1200 Bp was observed for *Plasmodium* genus on day 0. All 270 subjects showed ∼120 Bp band in the nested PCR, which is indicative of *P. vivax* malaria (Fig. 2). Six (2.2%) patients demonstrated 120 bp bands in nested PCR on day 5. On days 7 and 28 no band was illustrated for *P. vivax*. Two patients with *vivax* malaria by microscopy assay showed mixed infection with P. *falciparum* that was removed from the study. Mean parasite clearance time (MPCT) and SD were 2.43 (95% confidence interval 2.31-2.5) and 0.8 days respectively. MPCT based on sex was 2.39 and 2.44 days in males and females, respectively. There was no significant difference regarding MPCT between males and females (T = 0.5, P = 0.59, df= 268). The number of parasite ranged from 1,000 to 35,000 P/ µl blood with geometric mean of 7,500 P/µl.

## Discussion

Emergence of chloroquine resistant *P. vivax* isolates in many countries especially in those near Iran and spread of chloroquine resistant *P. falciparum* in various areas of Iran mad it important to monitor closely of susceptibility of *P. vivax* to this drug in the country.

No genetic marker has been determined yet for chloroquine resistant *P. vivax* in contrary to *P. falciparum*, in vivo study, so, was performed to determine any resistance to chloroquine. The nested PCR could well detect low parasitemia so that, six patients exhibited *P. vivax* infection in contrary microscopy results on day 5. The findings of this study showed susceptibility of *P. vivax* to chloroquine in the region. This result is compatible with former studies in Iran (17, 20). It seems that decreased use of chloroquine in the area due to administration of S/P and ACT for treating *P. falciparum* as well as substantially decreased numbers of malaria infection play important roles in the observed susceptibility of *P. vivax* to chloroquine in this region.

Our results are in agreement with conducted studies in Pakistan (28) and Afghanistan (13), where *P. vivax* is susceptible to chloroquine. It seems there may be a regional pattern in *P. vivax* susceptibility to chloroquine due to traveling of people between this part of Iran and the neighbor countries. The nested PCR on day zero showed malaria infection in all samples, therefore the use of this diagnosis method could be applied to in vivo studies. In addition, parasitological and molecular investigations indicated no recurrent infection on days 7 and 28. In the study conducted in Turkey by PCR, 9.5% resistance in *P. vivax* isolates to chloroquine was reported (6), while no case of resistance has been observed in Iran by this method. In two previous studies performed in southeast of Iran, mean parasite clearance time has been reported as 1.95 and 2.56 days, respectively (17, 20). Our findings indicate MPCT was 2.4 days, which is higher than that of Edrissian (1999) study but is compatible with Nateghpour (2007) study. It seems the presence of various haplotypes of *P. vivax* in the area exhibited different parasite clearance time in our study. Otherwise, the patients may have been infected by haplotypes with different susceptibility to chloroquine. No association was determined between the level of parasitemia, sex, and age with parasite clearance time in the patients.

In conclusion, *P. vivax* still has suitable susceptibility to chloroquine in southeast of Iran and chloroquine can be administrated for treatment of the *vivax* malaria patients at this region. Nested PCR was also suitable assay to determine exact malaria parasite clearance time in our *in vivo* study.
